# Exploring the Clinical Utility of Osteoprotegerin in Heart Failure—A Systematic Review and Meta-Analysis

**DOI:** 10.3390/ijms262211053

**Published:** 2025-11-15

**Authors:** Gifar Gazi, Gabi Gazi, Robert Cristian Cruciat, Daniel-Corneliu Leucuta, Stefan-Lucian Popa, Abdulrahman Ismaiel

**Affiliations:** 1 Faculty of Medicine, “Iuliu Hatieganu” University of Medicine and Pharmacy, 400006 Cluj-Napoca, Romania; gifargazzi@gmail.com (G.G.); gabigazi99@gmail.com (G.G.); robert.cruciat10@gmail.com (R.C.C.); 2 Department of Medical Informatics and Biostatistics, “Iuliu Hatieganu” University of Medicine and Pharmacy, 400349 Cluj-Napoca, Romania; 3 2nd Department of Internal Medicine, “Iuliu Hatieganu” University of Medicine and Pharmacy, 400006 Cluj-Napoca, Romania; popa.stefan@umfcluj.ro (S.-L.P.); abdulrahman.ismaiel@yahoo.com (A.I.)

**Keywords:** heart failure, osteoprotegerin, biomarkers, NYHA classification, meta-analysis

## Abstract

Osteoprotegerin (OPG) is a glycoprotein involved in bone metabolism and cardiovascular health, with emerging evidence suggesting its role in heart failure (HF). Despite its potential as a biomarker, the association between circulating OPG levels and HF severity remains unclear. This systematic review and meta-analysis aimed to evaluate OPG levels in HF patients and their relationship with disease severity according to the New York Heart Association (NYHA) classification. A comprehensive search of PubMed, EMBASE, and Scopus was conducted to identify observational studies assessing OPG levels in HF patients. Studies were included if they reported OPG levels in HF patients and controls, with subgroup analyses according to NYHA classification when available. Risk of bias assessment was performed using the Newcastle–Ottawa Scale (NOS). The principal outcome was the mean difference (MD) in circulating OPG levels between HF patients and controls. Random-effects meta-analysis models were used to pool the data. Thirteen studies with a total of 1387 participants were included in the quantitative and qualitative synthesis. Overall, OPG levels were significantly elevated in HF patients compared to healthy controls (2.490 [95% CI 0.531, 4.449]). Subgroup analysis showed a significant decrease in OPG levels in controls versus NYHA II patients (−1.503 [95% CI −2.402, −0.604]). However, no statistically significant difference was found when comparing OPG levels between the combined NYHA II/III group and controls (−1.019 [95% CI −2.451, 0.412]). OPG levels are significantly elevated in HF patients compared to controls, with a progressive increase in NYHA II patients. However, the lack of significance in the NYHA II/III group highlights the need for further studies with a more comprehensive NYHA classification breakdown.

## 1. Introduction

Heart failure (HF) remains a leading cause of morbidity and mortality worldwide, with an estimated 64 million people affected globally [1]. Despite major advances in cardiovascular care, the burden of HF continues to rise, driven by aging populations and the increasing prevalence of comorbid conditions such as hypertension, diabetes mellitus, and obesity [2]. The condition imposes a significant economic strain on healthcare systems, with frequent hospitalizations and complex long-term management contributing to high costs and reduced quality of life for patients.

The diagnosis of HF is based on a combination of clinical history, physical examination, and imaging techniques, most notably transthoracic echocardiography for evaluating left ventricular function and structural abnormalities. Blood tests also play a pivotal role in the diagnostic algorithm, particularly natriuretic peptides such as B-type natriuretic peptide (BNP) and N-terminal proBNP (NT-proBNP), which are recommended in current guidelines to support the diagnosis, assess prognosis, and guide therapy. However, these markers have limitations, including variable levels influenced by age, body mass index, renal function, and comorbidities, highlighting the need for novel and complementary biomarkers that can enhance diagnostic accuracy and provide additional pathophysiological insights.

In recent years, there has been a growing interest in the role of biomarkers reflecting myocardial stress, inflammation, fibrosis, and remodeling in HF. Several recent reviews support this broad biomarker expansion in HF management and research [3]. Among these, osteoprotegerin (OPG) is a soluble glycoprotein of the tumor-necrosis-factor receptor superfamily that functions primarily as a decoy receptor for RANKL (receptor activator of nuclear factor κB ligand) and binds TRAIL (TNF-related apoptosis-inducing ligand) [4,5]. OPG is produced by osteoblasts, vascular smooth muscle cells, endothelial cells, adipocytes and immune cells, and while it is best known for inhibiting osteoclast differentiation and bone resorption, growing evidence implicates OPG in cardiovascular biology [4,5]. Mechanistically, elevated OPG may reflect and mediate chronic vascular inflammation, endothelial dysfunction, and vascular calcification; it has been linked to matrix-metalloproteinase activation, adverse ventricular remodeling, and impaired myocardial function in both experimental models and clinical cohorts. Several recent reviews and cohort studies have reported associations between higher circulating OPG and adverse cardiac structure, diastolic dysfunction, and worse clinical outcomes (mortality, HF hospitalization), supporting its candidacy as a biomarker that captures inflammation- and remodeling-related pathways complementary to natriuretic peptides [4,5].

Beyond natriuretic peptides, which remain the most widely used biomarkers in HF, several other circulating molecules have been investigated for their potential to improve diagnostic precision and risk stratification. These include markers of myocardial injury (e.g., troponins) [6], extracellular matrix remodeling (e.g., galectin-3, ST2) [7,8], and vascular dysfunction. Adrenomodulin (ADM), a vasodilatory peptide with roles in endothelial barrier function and fluid homeostasis, has recently gained attention as a promising biomarker in this context. Elevated levels of ADM have been associated with worse outcomes in HF patients and may offer additive prognostic value when used alongside established markers such as NT-proBNP [9].

Emerging evidence suggests that OPG may play a pathophysiological role in the development and progression of HF [10]. Elevated circulating OPG levels have been observed in patients with various cardiovascular diseases, including atherosclerosis, coronary artery disease, and left ventricular dysfunction. Several clinical studies have reported associations between higher OPG concentrations and adverse outcomes in HF populations, including increased mortality, rehospitalization, and ventricular remodeling. Nevertheless, findings have been heterogeneous, and the strength of these associations remains uncertain across different patient populations and study designs.

Given the growing body of literature investigating OPG in HF, this systematic review and meta-analysis aims to synthesize current evidence on the role of circulating OPG levels in patients with HF. Specifically, we seek to assess the difference between OPG levels in HF patients compared to controls, as well as according to NYHA severity classes. By consolidating existing data, this study aims to clarify the clinical utility of OPG as a biomarker in the context of HF and guide future research directions.

## 2. Materials and Methods

This systematic review and meta-analysis were written as per the Preferred Reporting Items for Systematic Reviews and Meta-Analyses (PRISMA) 2020 statement [11].

### 2.1. Data Source and Strategy

A comprehensive electronic search was performed across PubMed, Embase, and Scopus to identify observational studies evaluating OPG levels in HF. The search strategy is detailed in Supplementary Material 1. Reference lists of included studies were manually screened to capture additional relevant publications. The search included all records up to 25 October 2024 and was performed independently by three investigators (Gi.G., R.C.C., and Ga.G.), with discrepancies resolved by discussion. No restrictions were applied for date, country, or language. Titles and abstracts were screened for eligibility, followed by full-text review based on predefined inclusion and exclusion criteria. Data extraction was independently performed by three investigators (Gi.G., R.C.C., and Ga.G.) and verified by a fourth (A.I.), with any conflicts resolved by consulting the original articles. Extracted variables included author, publication year, country, study design, population characteristics, sample size, proportion of heart failure patients, sex, mean age, body mass index (BMI), sex distribution, OPG levels reported as mean ± SD or median (interquartile range), and main study outcome, which were collated and presented in the manuscript text.

### 2.2. Eligibility Criteria

The inclusion criteria for original articles in this systematic review and meta-analysis were as follows: observational cohort, cross-sectional, or case–control studies that assessed OPG in the context of HF were considered eligible. HF had to be diagnosed according to the criteria specified within each individual study. Only human studies were included, with no restrictions on participants’ sex, race, or ethnicity. Studies were excluded if they did not report data on OPG in relation to HF and control subjects or the NYHA classification. Additionally, editorials, letters, short surveys, commentaries, case reports, conference abstracts, review articles, pediatric studies, practice guidelines, and abstracts without accompanying full-text articles were excluded.

### 2.3. Risk of Bias Assessment in Individual Studies

The investigators (Gi.G., R.C.C. and Ga.G.) independently applied the NOS [12,13], to evaluate risk of bias and internal validity of the included studies. Any discrepancies in quality ratings were resolved through discussion. Separate assessment forms were used for case–control and cross-sectional studies. Each study was scored based on stars received across selection, comparability, and outcome domains, with total scores ranging from 0 to 10. Studies with ≥7 stars were considered high-quality. The quality assessment did not influence study eligibility.

### 2.4. Summary Measures and Synthesis of Results

Data analysis for the systematic review and meta-analysis was conducted using the R software environment (https://www.jstatsoft.org/article/view/v049i05, accessed on 25 November 2024), utilizing the Metafor package through OpenMeta Analyst to perform all statistical evaluations and model estimations [14,15]. The principal summary outcome was the mean difference (MD) of OPG levels in HF patients compared to controls. Heterogeneity among studies was assessed using the χ^2^-based Q test together with the I^2^ statistic. In accordance with the Cochrane Handbook guidelines for evaluating heterogeneity, I^2^ values were interpreted as follows: 0–40% indicating low or negligible heterogeneity, 30–60% representing moderate heterogeneity, 50–90% reflecting substantial heterogeneity, and 75–100% suggesting considerable heterogeneity [16]. For studies that reported the outcomes as the medians with IQRs, corresponding means and standard deviation (SD) were estimated to ensure data comparability across analyses. In studies that included multiple subgroups of OPG patients or control participants, group-level means and standard deviations were aggregated to calculate values representing the full cohort, following guidance from the Cochrane Handbook. Subgroup analyses were then performed based on HF severity according to NYHA classification, using the data available from the extracted study results. All meta-analyses were conducted using random-effects models with restricted maximum likelihood estimation. For each included study, the outcomes were reported as the estimated MD with corresponding 95% CI, specifying the lower and upper bounds, standard error, and *p*-value. Statistical significance was defined as a *p*-value below 0.05. Analyses were carried out only when a minimum of two studies provided the same outcome, either as mean and SD or as median with IQR, along with their lower and upper CI limits.

## 3. Results

### 3.1. General Results

**Figure 1:**
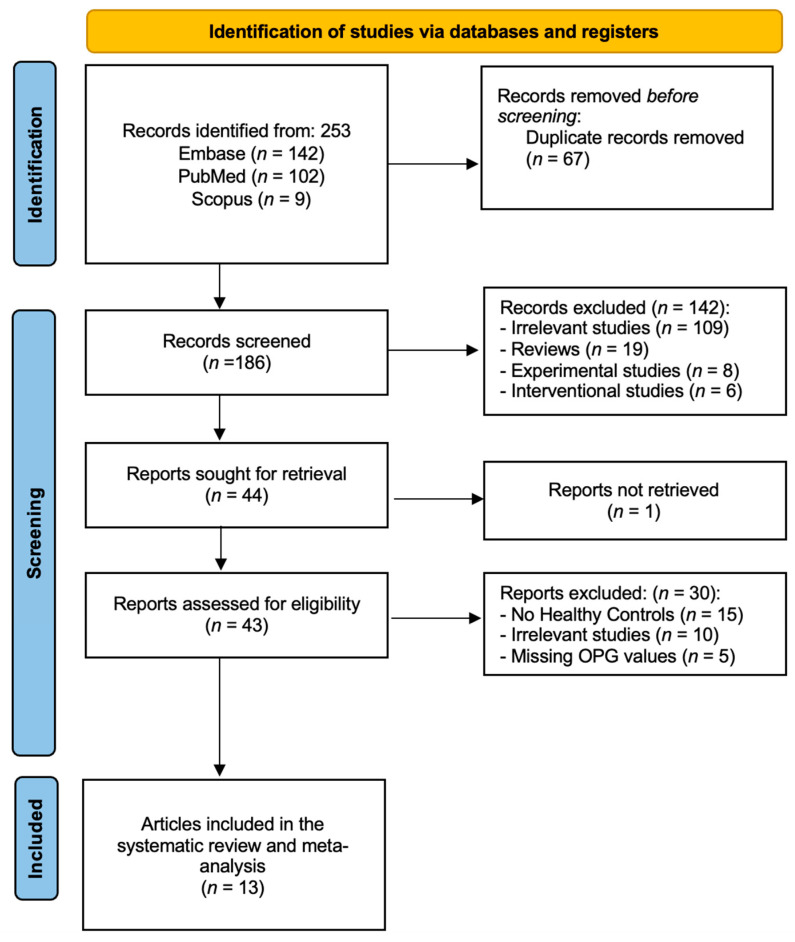
PRISMA flow diagram—identification, screening, and inclusion phases of our systematic review and meta-analysis.

As illustrated in Figure 1, the initial search generated two hundred fifty-three publications (102 articles from PubMed, 142 from EMBASE, and 9 from Scopus). Sixty-seven studies were identified as duplicates and eliminated. After deleting duplicates, one hundred eighty-six papers were assessed for inclusion and exclusion criteria fulfillment using the titles and abstracts. Following the initial screening, a total of one hundred and forty-two articles has been excluded as follows: (1) one hundred and nine irrelevant studies, (2) nineteen reviews, (3) eight experimental studies, and (4) six interventional studies. We were unable to retrieve single article. Following that, we thoroughly reviewed and evaluated the full texts for the remaining 43 articles to determine their eligibility. 32 of the articles were excluded for various reasons. Fifteen articles reported no healthy controls [17,18,19,20,21,22,23,24,25,26,27,28,29,30,31]. Ten articles were considered irrelevant [32,33,34,35,36,37,38,39,40,41] and five articles reported no OPG values [42,43,44,45,46]. The total number of articles included in the qualitative synthesis was thirteen studies, out of which all were included in the quantitative synthesis [47,48,49,50,51,52,53,54,55,56,57,58,59].

### 3.2. Study Characteristics

A summary of the main characteristics of the included studies is presented in Supplementary Table S1. This systematic review and meta-analysis included a total number of 1387 individuals. According to studies that reported sex distribution, males presented a larger proportion of the included participants (males—890 [64.2%], females—497 [35.8%]), while mentioning that one study did not report sex distribution [53]. HF was present in 808 subjects (58.3%) of the total study population. Twelve studies were conducted in Europe (Germany *n* = 1, Finland *n* = 1, Greece *n* = 1, Italy *n* = 2, Ukraine *n* = 1, Poland *n* = 1, Romania *n* = 1, Serbia *n* = 3, Croatia *n* = 1) and one in Asia (Korea *n* = 1)

### 3.3. Definition of HF

HF was assessed using echocardiography for diagnosis in most studies (*n* = 9) [47,48,49,50,51,55,56,57,59], while two studies did not specify their diagnostic modality [52,53]. Moreover, most of the studies used echocardiography together with additional methods, including blood tests, MRI, scintigraphy with Technetium-99, angiography, and cardiac catheterization.

### 3.4. OPG Levels in HF

#### 3.4.1. OPG Levels in HF Patients vs. Controls

**Figure 2:**
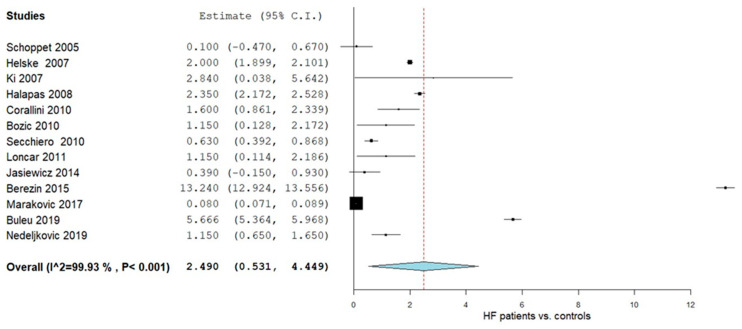
OPG levels in HF patients vs. controls [47,48,49,50,51,52,53,54,55,56,57,58,59].

OPG levels were evaluated in a total of thirteen studies comparing values in HF patients with control subjects [47,48,49,50,51,52,53,54,55,56,57,58,59]. Figure 2 summarizes the obtained meta-analysis results. The pooled analysis that assessed OPG levels in adult HF patients vs. control subjects showed an overall MD of 2.490 (95% CI 0.531, 4.449). Considerable heterogeneity was reported with an I^2^ = 99.93% and a *p*-value < 0.001.

#### 3.4.2. Controls vs. NYHA Class II

**Figure 3:**
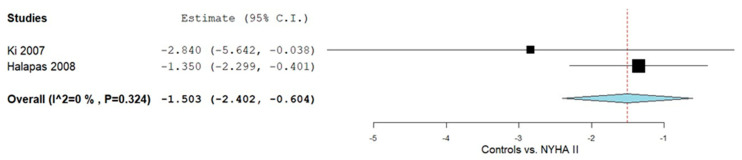
OPG levels in HF patients according to controls vs. NYHA II classification II [49,50].

Subgroup analyses in adults were conducted to assess OPG levels across NYHA classifications relative to controls (Figure 3). For NYHA II patients, two studies [49,50] provided relevant data. The pooled MD was −1.503 (95% CI: −2.402 to −0.604), with heterogeneity deemed negligible (I^2^ = 0%, *p* = 0.324)

#### 3.4.3. Controls vs. NYHA Class II and III

**Figure 4:**
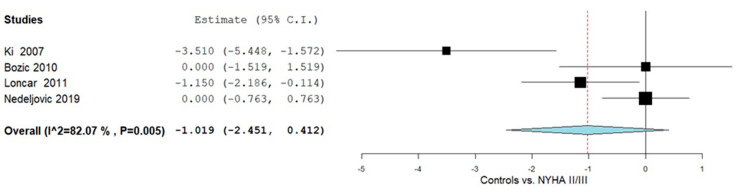
OPG levels in HF patients according to controls vs. NYHA II/III [49,51,54,57].

Further subgroup analyses were performed in adults to compare OPG levels between NYHA II/III HF patients and controls, as illustrated in Figure 4. Four studies [49,51,54,57] reported OPG measurements for this comparison. The pooled MD was −1.019 (95% CI: −2.451 to 0.412), indicating lower OPG levels in the patient group, accompanied by considerable heterogeneity across studies (I^2^ = 82.07%, *p* = 0.005).

### 3.5. Quality Assessment

The methodological quality of studies included in the systematic review and meta-analysis was assessed using NOS [12,13]. Thirteen cross-sectional studies were evaluated (Supplementary Table S2): 10/10 (*n* = 2), 9/10 (*n* = 2), 8/10 (*n* = 6), and 6/10 (*n* = 3). All studies clearly stated research objectives. Nearly half had samples that were truly or somewhat representative of the target population, with adequate and justified sample sizes. All studies employed validated measurement tools. Three studies controlled for the primary confounder and at least one additional factor. Outcomes were consistently assessed via record linkage, appropriate statistical tests were applied, and results were reported clearly.

## 4. Discussion

HF remains a critical global health challenge, with rising incidence and substantial morbidity and mortality despite ongoing advancements in diagnosis and treatment. In this context, the identification of novel biomarkers capable of enhancing risk stratification and capturing underlying pathophysiological processes is of paramount importance. This systematic review and meta-analysis provide a comprehensive synthesis of current evidence regarding circulating OPG levels in HF patients. We included thirteen articles with a total population of 1387 in our quantitative and qualitative synthesis. Our findings demonstrate that OPG levels are significantly elevated in individuals with HF compared to healthy controls, with a notable increase observed in patients classified as NYHA Class II. However, when NYHA Class II and III patients were analyzed together, the difference in OPG levels compared to controls did not reach statistical significance. These results suggest a potential role for OPG as a marker of early HF severity, although further data are needed to clarify its performance across the entire NYHA spectrum.

OPG has been recently increasingly recognized as a critical mediator linking the immune system to cardiovascular pathology. Expressed by various cell types, including immune cells, endothelial cells, and vascular smooth muscle cells, OPG modulates inflammatory responses by acting as a decoy receptor for ligands such as RANKL and TRAIL. In conditions marked by chronic inflammation, such as HF, elevated OPG levels may signal persistent immune activation. This heightened inflammatory state can drive endothelial dysfunction and vascular calcification, ultimately contributing to maladaptive cardiac remodeling and impaired myocardial function. Thus, the interplay between OPG and the immune system not only reflects ongoing inflammatory processes but also may play a direct role in the development and progression of HF [60,61].

Multiple studies have underscored the potential role of OPG in the development and progression of HF [62]. Ueland et al. demonstrated persistently elevated OPG expression in both rat and human HF models, particularly within left ventricular cardiomyocytes, with levels correlating strongly with disease severity [63]. The same group also highlighted the involvement of the OPG/RANK/RANKL axis in HF pathogenesis, suggesting that RANKL-induced matrix metalloproteinase activity may contribute to left ventricular dysfunction and remodeling. Similarly, Omland et al. found that higher OPG levels in the general population were independently associated with adverse cardiac structural and functional parameters, such as increased left ventricular mass and reduced ejection fraction [64]. Supporting these observations, the GISSI-HF trial showed that elevated circulating OPG was a strong, independent predictor of mortality and cardiovascular hospitalization in chronic HF patients, emphasizing its potential utility as a biomarker for risk stratification and a possible therapeutic target [28].

Our findings are largely consistent with previously published literature, which has reported elevated OPG concentrations in patients with cardiovascular diseases, including HF. In a study published by Frioes et al., serum OPG levels at discharge were identified as a significant prognostic marker in patients with acute HF. The prospective study demonstrated a direct association between elevated OPG concentrations and an increased risk of all-cause mortality or hospital readmission within six months, independent of established prognostic indicators. A graded risk increase was observed across OPG quartiles, with the highest quartile exhibiting a 2.44-fold greater risk, reinforcing the potential of OPG as a biomarker for adverse outcomes in both preserved and reduced ejection fraction acute HF [65].

We evaluated OPG levels in HF patients and reported that OPG levels are significantly increased in adult HF patients, especially when compared to healthy controls. Additionally, the subgroup analysis in our study further revealed that OPG levels were significantly elevated in NYHA Class II patients compared to controls, but the combined NYHA II/III group did not show a statistically significant difference. This result may be influenced by the limited availability of studies reporting OPG values according to NYHA classification. Due to these constraints, we were only able to include two studies [49,50] in this analysis, which may have impacted the statistical power needed to detect a meaningful difference. While our findings suggest no significant increase in OPG levels in NYHA II/III versus controls, the limited data prevents definitive conclusions. Further studies with larger sample sizes and detailed NYHA stratification are necessary to better understand the relationship between OPG and HF severity, particularly in the NYHA II/III category.

From a clinical perspective, our findings suggest that OPG has potential utility as a biomarker in HF, particularly in early stages of disease. The observed elevation of OPG in NYHA Class II patients may reflect early myocardial stress and vascular inflammation, which precede overt hemodynamic compromise. If validated in larger, prospective cohorts, OPG could complement natriuretic peptides by providing additional information about vascular and remodeling-related pathways in HF pathogenesis. Moreover, OPG might serve as a candidate for targeted therapeutic monitoring, especially in patients at intermediate risk. However, given the current limitations in the evidence base, OPG cannot yet be recommended for routine clinical use in HF management.

Another possible explanation is that circulating OPG may not increase further in advanced HF because the cell types that normally produce OPG (e.g., endothelial and vascular smooth muscle cells) may become dysfunctional or depleted as the disease progresses. In such a scenario, worsening HF would not necessarily translate into higher serum OPG. This mechanism is biologically plausible, but the studies included in our review did not investigate cellular OPG production, so this concept remains hypothetical and requires future mechanistic research [66,67].

Several limitations of this systematic review and meta-analysis on OPG as a biomarker for HF should be acknowledged. First, the overall number of included studies and participants was relatively modest, particularly within subgroup analyses stratified by NYHA classification, which may limit the statistical power and generalizability of our findings. Second, substantial heterogeneity was observed across studies, likely stemming from differences in study design, sample size, HF etiology, comorbid conditions, and methods used to measure OPG. Third, most studies relied on a single baseline measurement of OPG without longitudinal follow-up, thereby limiting our ability to explore temporal trends, disease progression, or causal inferences. Moreover, methodological variability in OPG quantification, such as the use of enzyme-linked immunosorbent assay (ELISA) versus radioimmunoassay (RIA), introduces potential inconsistencies due to differences in assay sensitivity, specificity, and susceptibility to measurement error. Lastly, the use of either serum or plasma OPG across studies may contribute to further variability, given known differences in biomarker concentrations between these biological matrices. Although current evidence supports the potential role of OPG as a biomarker in HF, several research gaps remain. Future studies should aim for larger, multicenter cohorts with standardized NYHA stratification to allow more robust subgroup analyses. OPG measurement techniques and biological matrices (serum vs. plasma) need standardized assay protocols and reference ranges. Longitudinal studies are required to clarify temporal dynamics of OPG and its predictive value for disease progression, hospitalization and mortality. Finally, mechanistic studies exploring the OPG/RANK/RANKL signaling axis in myocardial remodeling could help establish causal links and identify potential therapeutic targets. Addressing these gaps will be essential to determine whether OPG can move from an exploratory biomarker to a clinically actionable tool in heart failure management.

Despite the aforementioned limitations, this study has several notable strengths. To our knowledge, it is the first systematic review and meta-analysis evaluating the association between OPG levels and HF, integrating data from diverse populations and clinical settings. Rigorous methodological approaches were employed, including a predefined search strategy, adherence to PRISMA guidelines, and independent data extraction and quality assessment by multiple reviewers to minimize bias. Furthermore, subgroup and sensitivity analyses were conducted to explore sources of heterogeneity and assess the robustness of the results. These methodological strengths support the reliability of our conclusions and reinforce the potential relevance of OPG as a biomarker in the context of HF diagnosis, prognosis, and pathophysiology.

## 5. Conclusions

In patients with HF, OPG levels were significantly elevated compared to controls, with a notable increase in NYHA II versus controls. However, no significant increase was observed in the combined NYHA II/III group compared to controls. Overall, OPG appears to be a promising biomarker reflecting early myocardial and vascular remodeling and may complement established markers like natriuretic peptides by providing insight into inflammatory and remodeling mechanisms in HF. Yet, current evidence is limited by small, heterogeneous studies and non-standardized measurement methods. Future large, multicenter, longitudinal studies with standardized assays are needed to validate its prognostic value and clarify the mechanistic role of the OPG/RANK/RANKL axis, which could ultimately inform risk stratification and therapeutic approaches in HF.

## Data Availability

The original contributions presented in this study are included in the article/Supplementary Material. Further inquiries can be directed to the corresponding author.

## Supplementary Materials

The following supporting information can be downloaded at: https://www.mdpi.com/article/10.3390/ijms262211053/s1.

## References

[1] Savarese G., Becher P.M., Lund L.H., Seferovic P., Rosano G.M.C., Coats A.J.S. (2022). Global burden of heart failure: A comprehensive and updated review of epidemiology. Cardiovasc. Res..

[2] Shahim B., Kapelios C.J., Savarese G., Lund L.H. (2023). Global Public Health Burden of Heart Failure: An Updated Review. Card. Fail. Rev..

[3] Berezin A.E., Berezin A.A. (2023). Biomarkers in Heart Failure: From Research to Clinical Practice. Ann. Lab. Med..

[4] Akhtar S.M.M., Ali A., Fareed A., Hasan J. (2024). Osteoprotegerin (OPG): A potential biomarker for adverse cardiovascular events in stable coronary artery disease. Health Sci. Rep..

[5] Suh S.H., Oh T.R., Choi H.S., Kim C.S., Bae E.H., Ma S.K., Oh K.-H., Jung J.Y., Hyun Y.Y., Kim S.W. (2024). Circulating osteoprotegerin as a cardiac biomarker for left ventricular diastolic dysfunction in patients with pre-dialysis chronic kidney disease: The KNOW-CKD study. Clin. Res. Cardiol..

[6] Gori M., Senni M., Metra M. (2017). High-Sensitive Cardiac Troponin for Prediction of Clinical Heart Failure. Circulation.

[7] Ho J.E., Liu C., Lyass A., Courchesne P., Pencina M.J., Vasan R.S., Larson M.G., Levy D. (2012). Galectin-3, a Marker of Cardiac Fibrosis, Predicts Incident Heart Failure in the Community. J. Am. Coll. Cardiol..

[8] Parikh R.H., Seliger S.L., Christenson R., Gottdiener J.S., Psaty B.M., deFilippi C.R. (2016). Soluble ST2 for Prediction of Heart Failure and Cardiovascular Death in an Elderly, Community-Dwelling Population. J. Am. Heart Assoc..

[9] Cruciat R.C., Gazi G., Ismaiel A., Leucuta D.-C., Al Srouji N., Popa S.-L., Ismaiel M., Ensar D., Dumitrascu D.L. (2025). Unlocking the potential of biomarkers: The promise of adrenomedullin and its precursors in diagnosing and assessing heart failure. Int. J. Cardiol..

[10] Kuku K.O., Oyetoro R., Hashemian M., Livinski A.A., Shearer J.J., Joo J., Psaty B.M., Levy D., Ganz P., Roger V.L. (2024). Proteomics for heart failure risk stratification: A systematic review. BMC Med..

[11] Page M.J., McKenzie J.E., Bossuyt P.M., Boutron I., Hoffmann T.C., Mulrow C.D., Shamseer L., Tetzlaff J.M., Akl E.A., Brennan S.E. (2021). The PRISMA 2020 statement: An updated guideline for reporting systematic reviews. BMJ (Clin. Res. ed.).

[12] Wells G., Shea B., O’Connell D., Peterson J., Welch V., Losos M., Tugwell P. (2000). The Newcastle–Ottawa Scale (NOS) for Assessing the Quality of Non-Randomized Studies in Meta-Analysis. https://www.ohri.ca/programs/clinical_epidemiology/oxford.asp.

[13] Lo C.K., Mertz D., Loeb M. (2014). Newcastle-Ottawa Scale: Comparing reviewers’ to authors’ assessments. BMC Med. Res. Methodol..

[14] Wallace B.C., Dahabreh I.J., Trikalinos T.A., Lau J., Trow P., Schmid C.H. (2012). Closing the Gap between Methodologists and End-Users: R as a Computational Back-End. J. Stat. Softw..

[15] Viechtbauer W. (2010). Conducting Meta-Analyses in R with the metafor Package. J. Stat. Softw..

[16] Akl E., Altman D., Aluko P., Beaton D., Berlin J., Bhaumik B., Bingham C., Boers M., Booth A., Boutron I. (2019). Cochrane Handbook for Systematic Reviews of Interventions.

[17] Chen Y.-H., Wu Y.-W., Yang W.-S., Wang S.-S., Lee C.-M., Chou N.-K., Hsu R.-B., Lin Y.-H., Lin M.-S., Ho Y.-L. (2012). Relationship between Bone Mineral Density and Serum Osteoprotegerin in Patients with Chronic Heart Failure. PLoS ONE.

[18] Erkol A., Oduncu V., Pala S., Kızılırmak F., Kılıcgedik A., Yılmaz F., Güler A., Karabay C.Y., Kırma C. (2012). Plasma osteoprotegerin level on admission is associated with no-reflow phenomenon after primary angioplasty and subsequent left ventricular remodeling in patients with acute ST-segment elevation myocardial infarction. Atherosclerosis.

[19] Erkol A., Pala S., Kırma C., Oduncu V., Dündar C., Izgi A., Tigen K., Gibson C.M. (2011). Relation of circulating osteoprotegerin levels on admission to microvascular obstruction after primary percutaneous coronary intervention. Am. J. Cardiol..

[20] Fuernau G., Zaehringer S., Eitel I., de Waha S., Droppa M., Desch S., Schuler G., Adams V., Thiele H. (2013). Osteoprotegerin in ST-elevation myocardial infarction: Prognostic impact and association with markers of myocardial damage by magnetic resonance imaging. Int. J. Cardiol..

[21] Gullestad L., Ueland T., Vinge L.E., Finsen A., Yndestad A., Aukrust P. (2012). Inflammatory cytokines in heart failure: Mediators and markers. Cardiology.

[22] Jansson A.M., Hartford M., Omland T., Karlsson T., Lindmarker P., Herlitz J., Ueland T., Aukrust P., Caidahl K. (2012). Multimarker risk assessment including osteoprotegerin and CXCL16 in acute coronary syndromes. Arterioscler. Thromb. Vasc. Biol..

[23] Leistner D.M., Seeger F.H., Fischer A., Röxe T., Klotsche J., Iekushi K., Seeger T., Assmus B., Honold J., Karakas M. (2012). Elevated levels of the mediator of catabolic bone remodeling RANKL in the bone marrow environment link chronic heart failure with osteoporosis. Circ. Heart Fail..

[24] Omland T., Drazner M.H., Ueland T., Abedin M., Murphy S.A., Aukrust P., de Lemos J.A. (2007). Plasma osteoprotegerin levels in the general population: Relation to indices of left ventricular structure and function. Hypertension.

[25] Omland T., Ueland T., Jansson A.M., Persson A., Karlsson T., Smith C., Herlitz J., Aukrust P., Hartford M., Caidahl K. (2008). Circulating osteoprotegerin levels and long-term prognosis in patients with acute coronary syndromes. J. Am. Coll. Cardiol..

[26] Pedersen E.R., Ueland T., Seifert R., Aukrust P., Schartum-Hansen H., Ebbing M., Bleie Ø., Igland J., Svingen G., Nordrehaug J.E. (2010). Serum osteoprotegerin levels and long-term prognosis in patients with stable angina pectoris. Atherosclerosis.

[27] Røysland R., Bonaca M.P., Omland T., Sabatine M., Murphy S.A., Scirica B.M., Bjerre M., Flyvbjerg A., Braunwald E., Morrow D.A. (2012). Osteoprotegerin and cardiovascular mortality in patients with non-ST elevation acute coronary syndromes. Heart.

[28] Røysland R., Masson S., Omland T., Milani V., Bjerre M., Flyvbjerg A., Di Tano G., Misuraca G., Maggioni A.P., Tognoni G. (2010). Prognostic value of osteoprotegerin in chronic heart failure: The GISSI-HF trial. Am. Heart J..

[29] Schnabel R.B., Larson M.G., Yamamoto J.F., Kathiresan S., Rong J., Levy D., Keaney J.F., Wang T.J., Vasan R.S., Benjamin E.J. (2009). Relation of multiple inflammatory biomarkers to incident atrial fibrillation. Am. J. Cardiol..

[30] Ueland T., Aukrust P., Dahl C.P., Husebye T., Solberg O.G., Tønnessen T., Aakhus S., Gullestad L. (2011). Osteoprotegerin levels predict mortality in patients with symptomatic aortic stenosis. J. Intern. Med..

[31] Ueland T., Dahl C.P., Kjekshus J., Hulthe J., Böhm M., Mach F., Goudev A., Lindberg M., Wikstrand J., Aukrust P. (2011). Osteoprotegerin predicts progression of chronic heart failure: Results from CORONA. Circ. Heart Fail..

[32] Balion C.M., Santaguida P., McKelvie R., Hill S.A., McQueen M.J., Worster A., Raina P.S. (2008). Physiological, pathological, pharmacological, biochemical and hematological factors affecting BNP and NT-proBNP. Clin. Biochem..

[33] Candido R., Toffoli B., Corallini F., Bernardi S., Zella D., Voltan R., Grill V., Celeghini C., Fabris B. (2010). Human full-length osteoprotegerin induces the proliferation of rodent vascular smooth muscle cells both in vitro and in vivo. J. Vasc. Res..

[34] Demyanets S., Huber K., Wojta J. (2011). Inflammation and the cardiovascular system. Eur. Surg..

[35] Feng W., Li W., Liu W., Wang F., Li Y., Yan W. (2009). IL-17 induces myocardial fibrosis and enhances RANKL/OPG and MMP/TIMP signaling in isoproterenol-induced heart failure. Exp. Mol. Pathol..

[36] Gruson D., Ahn S.A., Rousseau M.F. (2011). Biomarkers of inflammation and cardiac remodeling: The quest of relevant companions for the risk stratification of heart failure patients is still ongoing. Biochem. Med. (Zagreb).

[37] Henkens M., van Ommen A.M., Remmelzwaal S., Valstar G.B., Wang P., Verdonschot J.A.J., Hazebroek M.R., Hofstra L., van Empel V.P.M., Beulens J.W.J. (2022). The HFA-PEFF score identifies ‘early-HFpEF’ phenogroups associated with distinct biomarker profiles. ESC Heart Fail..

[38] Koo H.M., Do H.M., Kim E.J., Lee M.J., Shin D.H., Kim S.J., Oh H.J., Yoo D.E., Kim J.K., Park J.T. (2011). Elevated osteoprotegerin is associated with inflammation, malnutrition and new onset cardiovascular events in peritoneal dialysis patients. Atherosclerosis.

[39] Pincott E.S., Burch M. (2011). New biomarkers in heart failure. Prog. Pediatr. Cardiol..

[40] Schlieper G., Westenfeld R., Brandenburg V., Ketteler M. (2007). Inhibitors of calcification in blood and urine. Semin. Dial..

[41] Vik A., Brodin E., Børvik T., Sveinbjørnsson B., Hansen J.B. (2006). Serum osteoprotegerin in young survivors of myocardial infarction. Thromb. Haemost..

[42] Gupta S., Drazner M.H., de Lemos J.A. (2009). Newer biomarkers in heart failure. Heart Fail. Clin..

[43] Manhenke C., Ørn S., von Haehling S., Wollert K.C., Ueland T., Aukrust P., Voors A.A., Squire I., Zannad F., Anker S.D. (2013). Clustering of 37 circulating biomarkers by exploratory factor analysis in patients following complicated acute myocardial infarction. Int. J. Cardiol..

[44] Siasos G., Oikonomou E., Maniatis K., Georgiopoulos G., Kokkou E., Tsigkou V., Zaromitidou M., Antonopoulos A., Vavuranakis M., Stefanadis C. (2018). Prognostic significance of arterial stiffness and osteoprotegerin in patients with stable coronary artery disease. Eur. J. Clin. Investig..

[45] Ueland T., Yndestad A., Dahl C.P., Gullestad L., Aukrust P. (2012). TNF revisited: Osteoprotegerin and TNF-related molecules in heart failure. Curr. Heart Fail. Rep..

[46] Yue H., Li W., Desnoyer R., Karnik S.S. (2010). Role of nuclear unphosphorylated STAT3 in angiotensin II type 1 receptor-induced cardiac hypertrophy. Cardiovasc. Res..

[47] Schoppet M., Ruppert V., Hofbauer L.C., Henser S., Al-Fakhri N., Christ M., Pankuweit S., Maisch B. (2005). TNF-related apoptosis-inducing ligand and its decoy receptor osteoprotegerin in nonischemic dilated cardiomyopathy. Biochem. Biophys. Res. Commun..

[48] Helske S., Kovanen P.T., Lindstedt K.A., Salmela K., Lommi J., Turto H., Werkkala K., Kupari M. (2007). Increased circulating concentrations and augmented myocardial extraction of osteoprotegerin in heart failure due to left ventricular pressure overload. Eur. J. Heart Fail..

[49] Suh S.H., Oh T.R., Choi H.S., Kim C.S., Oh K.H., Lee J., Oh Y.K., Jung J.Y., Choi K.H., Ma S.K. (2021). Association of Circulating Osteoprotegerin Level with Blood Pressure Variability in Patients with Chronic Kidney Disease. J. Clin. Med..

[50] Halapas A., Zacharoulis A., Theocharis S., Karavidas A., Korres D., Papadopoulos K., Katopodis H., Stavropoulou A., Lembessis P., Xiromeritis C. (2008). Serum levels of the osteoprotegerin, receptor activator of nuclear factor kappa-B ligand, metalloproteinase-1 (MMP-1) and tissue inhibitors of MMP-1 levels are increased in men 6 months after acute myocardial infarction. Clin. Chem. Lab. Med..

[51] Bozic B., Loncar G., Prodanovic N., Radojicic Z., Cvorovic V., Dimkovic S., Popovic-Brkic V. (2010). Relationship between high circulating adiponectin with bone mineral density and bone metabolism in elderly males with chronic heart failure. J. Card. Fail..

[52] Corallini F., Secchiero P., Beltrami A.P., Cesselli D., Puppato E., Ferrari R., Beltrami C.A., Zauli G. (2010). TNF-alpha modulates the migratory response of mesenchymal stem cells to TRAIL. Cell. Mol. Life Sci. CMLS.

[53] Secchiero P., Corallini F., Beltrami A.P., Ceconi C., Bonasia V., Di Chiara A., Ferrari R., Zauli G. (2010). An imbalanced OPG/TRAIL ratio is associated to severe acute myocardial infarction. Atherosclerosis.

[54] Loncar G., Bozic B., Dimkovic S., Prodanovic N., Radojicic Z., Cvorovic V., Putnikovic B., Popovic V. (2011). Association of increased parathyroid hormone with neuroendocrine activation and endothelial dysfunction in elderly men with heart failure. J. Endocrinol. Investig..

[55] Jasiewicz M., Knapp M., Waszkiewicz E., Musiał W.J., Kamiński K.A. (2014). Potential pathogenic role of soluble receptor activator of nuclear factor-ĸB ligand and osteoprotegerin in patients with pulmonary arterial hypertension. Pol. Arch. Med. Wewn..

[56] Berezin A.E., Kremzer A.A., Berezina T.A., Martovitskaya Y.V. (2015). Pattern of circulating microparticles in chronic heart failure patients with metabolic syndrome: Relevance to neurohumoral and inflammatory activation. BBA Clin..

[57] Makarović S., Makarović Z., Bilić-Ćurčić I., Milas-Ahić J., Mihaljević I., Franceschi M., Jukić T. (2017). Serum Osteoprotegerin in Patients with Calcified Aortic Valve Stenosis in Relation to Heart Failure. Acta Clin. Croat..

[58] Buleu F.N., Luca C.T., Tudor A., Badalica-Petrescu M., Caraba A., Pah A., Georgescu D., Christodorescu R., Dragan S. (2019). Correlations between Vascular Stiffness Indicators, OPG, and 25-OH Vitamin D3 Status in Heart Failure Patients. Medicina.

[59] Nedeljkovic B.B., Loncar G., Vizin T., Radojicic Z., Brkic V.P., Kos J. (2019). Relationship of High Circulating Cystatin C to Biochemical Markers of Bone Turnover and Bone Mineral Density in Elderly Males with a Chronic Heart Failure. J. Med. Biochem..

[60] Montagnana M., Lippi G., Danese E., Guidi G.C. (2013). The role of osteoprotegerin in cardiovascular disease. Ann. Med..

[61] Dutka M., Bobiński R., Wojakowski W., Francuz T., Pająk C., Zimmer K. (2022). Osteoprotegerin and RANKL-RANK-OPG-TRAIL signalling axis in heart failure and other cardiovascular diseases. Heart Fail. Rev..

[62] Kamimura D., Buckley L., Claggett B., Yu B., Coresh J., Matsushita K., Chang P., Hoogeveen R., Ballantyne C., Hall M. (2021). Abstract 10769: Osteoprotegerin and Incident Heart Failure: The Atherosclerosis Risk in Communities Study. Circulation.

[63] Ueland T., Yndestad A., Øie E., Florholmen G., Halvorsen B., Frøland S.S., Simonsen S., Christensen G., Gullestad L., Aukrust P. (2005). Dysregulated osteoprotegerin/RANK ligand/RANK axis in clinical and experimental heart failure. Circulation.

[64] Ma T., Zhao J., Yan Y., Liu J., Zang J., Zhang Y., Ruan K., Xu H., He W. (2023). Plasma osteoprotegerin predicts adverse cardiovascular events in stable coronary artery disease: The PEACE trial. Front. Cardiovasc. Med..

[65] Friões F., Laszczynska O., Almeida P.B., Silva N., Guimarães J.T., Omland T., Azevedo A., Bettencourt P. (2015). Prognostic Value of Osteoprotegerin in Acute Heart Failure. Can. J. Cardiol..

[66] Giannitsi S., Bougiakli M., Bechlioulis A., Naka K. (2019). Endothelial dysfunction and heart failure: A review of the existing bibliography with emphasis on flow mediated dilation. JRSM Cardiovasc. Dis..

[67] Yin Z., Zhang J., Shen Z., Qin J.J., Wan J., Wang M. (2024). Regulated vascular smooth muscle cell death in vascular diseases. Cell Prolif..

