# Supplementary material for: Exploring the Clinical Utility of Osteoprotegerin in Heart Failure—A Systematic Review and Meta-Analysis
Source: Int J Mol Sci. 2025 Nov 15;26(22):11053. doi: 10.3390/ijms262211053 (PMC12653011; doi:10.3390/ijms262211053)
Supplement: Supplementary file 1 [file ijms-26-11053-s001.zip › Supplementary Material S1 Search strategy.pdf]

Supplementary Material S1

Search strategy

25.10.2024

PubMed: 102 articles

(("Osteoprotegerin"[Mesh]) OR ("Osteoprotegerin"[All Fields])) AND (("Heart Failure"[Mesh]) OR ("Heart Failure"[All Fields]))

Scopus: title, abstract, and keywords – 9 articles

(("Osteoprotegerin"[Mesh]) OR ("Osteoprotegerin"[All Fields])) AND (("Heart Failure"[Mesh]) OR ("Heart Failure"[All Fields]))

EMBASE: initial search 348 articles, applying filters – articles and articles in press – 142 articles

('Osteoprotegerin'/exp OR 'Osteoprotegerin') AND ('Heart Failure'/exp OR 'Heart Failure') AND ('article'/it OR 'article in press'/it)
