# Supplementary material for: Exploring the Clinical Utility of Osteoprotegerin in Heart Failure—A Systematic Review and Meta-Analysis
Source: Int J Mol Sci. 2025 Nov 15;26(22):11053. doi: 10.3390/ijms262211053 (PMC12653011; doi:10.3390/ijms262211053)
Supplement: Supplementary file 1 [file ijms-26-11053-s001.zip › Supplementary Table S1 OPG in HF Final.pdf]

**Supplementary Table S1.** Included studies evaluating the role of osteoprotegerin in heart failure			
First Author / Year / Country	Study Design	Study Characteristics	Main Findings
Schoppet et al. /2005/ Germany	Cohort Observational Study	• **Total Subjects**: 191<br>• **Population**: German Patients with HF<br>• **HF**: 105 (54,97%)<br>• **Mean age (years)**: HF: 50 ± 14 Controls: 50 ± 16<br>• **Sex (males)**: 191 (100%)<br>• **BMI**: Controls: 26.5 ± 3.3, HF: 27.5±4.5<br>• **Osteopretegrin Measurement Method:** ELISA<br>• **Plasma Osteoprotegerin (pmol/L) - Mean ± SD**: Controls (n=86): 3.6±2.0; HF (n=105): 3.7 ± 2.0<br>• **Osteopretegrin – AUC**: -<br>• **Measurement SERUM/PLASMA**: Plasma Osteoprotegrin	TRAIL levels were elevated in nonischemic DCM patients and correlated with heart dilation, while OPG levels were unchanged. TRAIL and OPG were present in heart tissue of DCM patients but not controls.
Helske et al. /2007/ Finland	Cohort Observational study	• **Total Subjects:** 61<br>• **Population:** Finish Patients with Aortic Stenosis<br>• **HF:** 49 (80,03%)<br>• Mean age (years): 68±10<br>• **Sex (males):** 64 (48,88%)<br>• **BMI: -**<br>• **Osteopretegrin Measurement Method:** ELISA<br>• **Plasma Osteoprotegerin (pmol/L) - Mean ± SD:** Controls (n=12): Aortic root: 2.5±0.1 Coronary sinus: 1.9±0.2 Femoral vein: 2.3±0.1; HF (n=49): Aortic root 5.4±0.4 Coronary Sinus: 5.0±0.4 Femoral vein: 4.3±0.3<br>• **Osteopretegrin – AUC: -**<br>• **Measurement SERUM/PLASMA:** Plasma Osteoprotegerin	Circulating OPG was elevated in AS patients with heart failure and decreased after valve replacement. OPG extraction by the heart and peripheral tissues was greater in heart failure, suggesting its involvement in heart failure pathogenesis
Ki et al. /2007/ Korea	Observational Study	• **Total Subjects:** 190<br>• **Population:** Korean Patients with HF<br>• **HF:** 127(66,84%)<br>• **Mean age (years)**: HF: 63±11: Controls: 56±8<br>• **Sex (males):** HF: 84 (66.14%); Controls: 32 (50.79)<br>• **BMI:-**<br>• **Osteopretegrin Measurement Method:** ELISA<br>• **Serum Osteopretegrin (pg/mL) - Mean IQR:** Controls (n=63): 549 IQR (466,632)**; HF** (n=127): 760 IQR (702,818). NYHA II: 683 (581,785); NYHA III: 768 (688,847); NYHA IV: 844 (694,993).<br>• **Osteopretegrin – AUC:-**<br>• **Measurement** SERUM/PLASMA**:** Serum Osteoprotegerin	Serum OPG levels were higher in patients with heart failure and acute myocardial infarction compared to healthy controls and increased with worsening NYHA classification. OPG levels were significantly associated with heart failure, AMI, and disease severity
Halapas et al./2008/ Greece	Prospective Observational study	• **Total Subjects**: 24<br>• **Population**: Greek patient with HF<br>• **HF**: 12 (50%)<br>• **Mean age (years)**: Controls: 53.8±4 HF: 53.8 ±3.7<br>• **Sex (males)**: 24(100%)<br>• **BMI:** Controls: -, HF:-<br>• **Osteopretegrin Measurement Method**: ELISA<br>• **Serum Osteoprotegerin (pmol/L) - Mean ± SD**: Controls (n=12): 1.91±0.16; HF NYHA II (n=12): 4.26±0.27<br>• **Osteopretegrin – AUC**: -.<br>• **Measurement SERUM/PLASMA**: Serum Osteoprotegrin	Serum levels of OPG, RANKL, MMP-1, and TIMP-1 were elevated in AMI patients, with significant correlations between RANKL and MMP-1/TIMP-1 six months post-MI. A low OPG/RANKL ratio suggested increased nuclear factor k-B signaling activity six months after MI, indicating a role in myocardial remodeling.

Corallini et al/2010/ Italy	Cohort Study	• **Total Subjects**: 61<br>• **Population**: Italian patients with Acute MI and HF<br>• **HF**: 21 (34,43%)<br>• **Mean age (years)**: Controls: 57.1 ± 9.4; HF: 66.6 ± 10.9<br>• **Sex (males)**: Controls: 26 (66.1%), HF: 14 (66.7%<br>• **BMI:** Controls: 23.9 ± 4.2, HF: 29.0 ± 4.8<br>• **Osteopretegrin Measurement Method**: ELISA<br>• **Serum Osteoprotegerin (pg/mL) - Mean ± SD**: Controls (n=40): 99.7 ± 36.3; HF (n=21): 195.6 ± 100<br>• **Osteopretegrin – AUC**: -.<br>• **Measurement SERUM/PLASMA**: Serum Osteoprotegrin	AMI patients who developed heart failure had lower circulating MSCs but higher TNF-a and OPG levels. TNF-a enhanced MSC migration via TRAIL but also increased OPG release, which inhibited TRAIL-induced MSC migration. This dual effect may impair MSC recruitment after AMI, contributing to heart failure development.
Bozic et al./2010/ Serbia	Cohort Observational study	• **Total Subjects:** 93<br>• **Population**: Serbian patients with HF<br>• **HF: 73** (78.49%)<br>• **Mean age (years):** HF 68±7; Controls: 67±7<br>• **Sex (males):** 73 (78.49%)<br>• **BMI:** HF: 28±5; Controls: 28±3<br>• **Osteopretegrin Measurement Method:** ELISA<br>• **Osteopretegrin (pmol/L)- Mean IQR**: Controls (n=20): Mean 10.8 IQR (63.5); HF NYHA II/III: 79.8 IQR (121.7)<br>• **Osteoprotegrin** – AUC: -<br>• **Measurement SERUM/PLASMA:** Serum Osteoprotegrin	In elderly males with chronic heart failure, serum adiponectin was elevated and positively correlated with bone turnover markers, including OPG, but was a negative predictor of BMD. Elevated OPG suggests increased bone remodeling, and adiponectin may negatively impact bone mass in CHF, indicating a link between bone and energy metabolism.
Secchiero et al./2010/ Italy	Cohort Observational study	• **Total Subjects:** 233<br>• **Population:** Italian Patients with HF<br>• **HF:** 113 (48.5%)<br>• **Mean age (years):** -<br>• **Sex (males)**:-<br>• **BMI:** -<br>• **Osteopretegrin Measurement Method:** ELISA<br>• **Serum Uromodulin (pg/mL) - Mean ± SD:** Controls (n=120): 1.62±0.58**;** HF (n=113): 2.25±1.16<br>• **Osteopretegrin – AUC:** -<br>• **Measurement SERUM/PLASMA:** Plasma Osteoprotegrin	In CAD patients, OPG levels were elevated while TRAIL levels were decreased after acute AMI, resulting in a higher OPG/TRAIL ratio. This ratio was even higher in AMI patients who developed heart failure, suggesting that an impaired OPG/TRAIL balance is linked to increased heart failure risk.
Loncar et al./2011/ Serbia	Observational study	• **Total Subjects:** 93<br>• **Population:** Serbian patients with HF<br>• **HF:** 73 (78,49%)<br>• **Mean age (years):** HF: 68±7, Controls: 67±7<br>• **Sex (males):** 93 (100%)<br>• **BMI:** Controls: 28±3**,** HF: 28±5<br>• **Osteopretegrin Measurement Method:** ELISA<br>• **Serum Osteoprotegerin (pg/ml) – Mean IQR:** Controls (n=20): 11 (65)**;** HF NYHA II/III IQR (n=73): 80 (122)<br>• **Osteoprotegrin – AUC:** 0.772 (0.652-0.891).<br>• **Measurement SERUM/PLASMA:** Serum Osteoprotegerin	In CHF, elevated PTH is linked to heart failure progression, endothelial dysfunction, and impaired quality of life. It correlates positively with NT-pro-BNP, adiponectin, and osteoprotegerin, but negatively with 25(OH)D, suggesting its potential as a monitoring marker.
Jasiewicz et al./2014/ Poland	Prospective Observational Study	• **Total Subjects:** 55<br>• **Population:** Polish patients with HF<br>• **HF: 24** (43,63%)<br>• **Mean age (years):** HF: 59.6 ±11.3, Controls: 49.9 ±13.5<br>• **Sex (males):** 34 (61.81%)<br>• **BMI:** Controls: 25.8 ±3.5**,** HF: 29.4 ±4<br>• **Osteopretegrin Measurement Method:** ELISA	sRANKL and OPG levels were elevated in PAH patients and correlated with disease severity and prognosis. Elevated OPG at baseline predicted clinical deterioration. sRANKL better discriminates between PAH and LVHF

		• **Serum Osteoprotegrin (pmol/L) - Mean ± SD:** Controls (n=31): 3.27 ±0.95**;** HF (n=24): 3.66 ±1.06<br>• **Osteopretegrin – AUC:-**.<br>• **Measurement** SERUM/PLASMA**:** Serum Osteoprotegrin	
Berezin et al./2015/ Ukraine	Retrospective Observational Study	• **Total Subjects:** 89<br>• **Population:** Ukrainian Patients with HF<br>• **HF:** 54 (60,67%)<br>• **Mean age (years):** HF: 48.42 ± 6.10, Controls: 46.12 ± 4.22<br>• **Sex (males):** HF: 34 (63.0%), Controls: 23 (65.7%)<br>• **BMI:** Controls: 21.5 (16.1–23.5)**,** HF: 28.5 (16.8–32.1)<br>• **Osteopretegrin Measurement Method:** ELISA<br>• **Serum Osteoprotegrin (pg/ml) - Mean ± SD:** Controls (n=35): 88.3 (37.5–136.6)**; HF** (n=105): 882.5 (697.1–1046.2)<br>• **Osteopretegrin – AUC:-**.<br>• **Measurement** SERUM/PLASMA**:** Serum Osteoprotegrin	OPG levels were significant predictors for a decreased CD62E+ to CD31+/annexin V+ ratio in MetS patients with CHF, highlighting its role in inflammation and biomechanical stress
Makarovic et al./2017/ Croatia	Pair-Matched Case-Control Study	• **Total Subjects:** 90<br>• **Population: Croatian** Patients with HF<br>• **HF: 29**(32.22%)<br>• **Mean age (years)**: HF:68.3725, Control: 66.7692<br>• **Sex (males):** 41 (45.56%)<br>• **BMI:** Controls: 26.65**,** HF: 28.376<br>• **Osteopretegrin Measurement Method:** ELISA<br>• **Serum Oteoprotegerin (pg/mL) - Mean** IQR**:** Controls (n=39): Median 4.89 IQR (3.88-5.65)**;** HF (n=29): Median 9.78 IQR (7.55-11.34)<br>• **Osteopretegrin – AUC:--**<br>• **Measurement SERUM/PLASMA:** Serum Osteoprotegrin	Serum OPG levels were higher in AS patients with heart failure, correlating with NT-proBNP, chest x-ray signs, aortic valve area, and left ventricular ejection fraction. OPG may be a useful biomarker for evaluating AS severity and guiding treatment decisions.
Buleu et al./2019/ Romania	Case-Control Study	• **Total Subjects:** 120<br>• **Population:** Romanian Patients with HF<br>• **HF:** 60 (50%)<br>• **Mean age (years):** HF: 68.43±9.00, Controls: 66.50±8.92<br>• **Sex (males):** HF: 30(50.00%), Controls: 29(48.33)<br>• **BMI:** Controls: 31.63±4.65**,** HF: 33.01±3.71<br>• **Osteopretegrin Measurement Method:** ELISA<br>• **Serum Osteoprotegrin (ng/ml) - Mean ± SD:** Controls (n=60): 1.3±0.67**; HF** (n=60): 4.7±0.25<br>• **Osteopretegrin – AUC:-**.<br>• **Measurement SERUM/PLASMA:** Serum Osteoprotegrin	In heart failure patients with reduced ejection fraction, elevated OPG and low 25-OH-D3 correlated with poor cardiac function and vascular stiffness. These markers, along with vitamin D status, may help assess disease severity and guide treatment like vitamin D supplementation.
Nedeljovic et al./2019/ Serbia	Prospective Cohort Study	• **Total Subjects:** 87<br>• **Population:** Serbian Patients with HF<br>• **HF:** 68 (78.16%)<br>• **Mean age (years):** HF: 68 ± 7, Controls: 67 ± 8<br>• **Sex (males):** 87(100%)<br>• **BMI:** Controls: 28 ± 3**,** HF: 27 ± 5<br>• **Osteopretegrin Measurement Method:** ELISA<br>• **Serum Osteoprotegrin (pg/mL) - Mean** IQR**:** NYHA II/III Controls (n=19): 10.8 (63.5) **;** HF (n=68): 79.8 (121.7) IQR<br>• **Osteopretegrin – AUC: -**.<br>• **Measurement** SERUM/PLASMA**:** Serum Osteoprotegrin	In males with CHF, Cystatin C was positively associated with osteoprotegerin, though details were lacking. Further research may clarify their role in bone loss and cardiovascular risk.
HF:heart failure; DCM:ilated Cardiomyopathy; OPG:Osteoprotegerin; AS: Aortic Stenosis; CHF:Congestive Heart Failure; ELISA: Enzyme-linked Immunosorbent Assay; PTH: Parathyroid Hormone; AMI: Acute Myocardial Infarction; TRAIL: TNF-related apoptosis inducing ligand; MI: Myocardial Infarction; BMD: Bone Mineral Density; CAD: Coronary-Artery Disease; PAH: Pulmonary artery Hypertension; MetS: Metabolic Syndrome;			
