# Supplementary material for: Exploring the Clinical Utility of Osteoprotegerin in Heart Failure—A Systematic Review and Meta-Analysis
Source: Int J Mol Sci. 2025 Nov 15;26(22):11053. doi: 10.3390/ijms262211053 (PMC12653011; doi:10.3390/ijms262211053)
Supplement: Supplementary file 1 [file ijms-26-11053-s001.zip › Supplementary Table S2 OPG.pdf]

Supplementary Table S2. The Newcastle-Ottawa Scale (NOS) for assessing the quality of cross-sectional studies

Study	Selection (Maximum 5 stars)				Comparability (Maximum 2 stars)	Outcome (Maximum 3 stars)		Score (Total maximum 10 stars)
	**Sample representativeness**	**Sample size**	**Non-Respondents**	**Ascertainment of the exposure (risk factor)**	**Comparability**	**Assessment of the outcome**	**Statistical test**	
Schoppet et al 2005	*	*	-	**	*	**	*	8
Helske et al 2007	*	*	-	**	*	*	*	8
Ki et al 2007	*	*	*	**	* *	**	*	10
Halapas et al 2008	-	-	-	**	*	**	*	6
Bozic et al 2010	*	*	-	**	*	**	*	8
Corallini et al 2010	-	-	-	**	*	**	*	6
Seccherio et al 2010	*	*	-	**	*	**	*	8
Loncar et al 2011	*	*	-	**	*	**	*	8
Jasiewicz et al 2014	-	-	-	**	*	**	*	6
Berezin et al 2015	*	*	*	**	*	**	*	9
Makarovic et al 2017	*	*	-	**	*	**	*	8
Buleu et al 2019	*	*	*	**	*	**	*	9
Nedeljkovic et al 2019	*	*	*	**	**	**	*	10
